# Total colonic aganglionosis: multicentre study of surgical treatment and patient‐reported outcomes up to adulthood

**DOI:** 10.1002/bjs5.50317

**Published:** 2020-07-13

**Authors:** P. Stenström, K. Kyrklund, M. Bräutigam, H. Engstrand Lilja, K. Juul Stensrud, A. Löf Granström, N. Qvist, L. Söndergaard Johansson, E. Arnbjörnsson, H. Borg, T. Wester, K. Björnland, M. P. Pakarinen

**Affiliations:** ^1^ Department of Paediatric Surgery, Children's Hospital in Lund Skane University Hospital Lund Lund Sweden; ^2^ Department of Paediatric Surgery, Queen Silvia's Children's Hospital Sahlgrenska University Hospital Gothenburg Gothenburg Sweden; ^3^ Department of Paediatric Surgery Uppsala University Children's Hospital Uppsala Sweden; ^4^ Division of Paediatric Surgery, Astrid Lindgren Children's Hospital Karolinska University Hospital, Karolinska Institutet Stockholm Sweden; ^5^ Department of Paediatric Surgery, Paediatric Research Centre, Children's Hospital Helsinki University Hospital Helsinki Finland; ^6^ Department of Paediatric Surgery Oslo University Hospital Oslo Norway; ^7^ Department of Paediatric Surgery Odense University Hospital, Research Unit Surgery, University of Southern Denmark Odense; ^8^ Department of Paediatric Surgery Rikshospitalet Copenhagen Copenhagen Denmark

## Abstract

**Background:**

Surgery for total colonic aganglionosis (TCA) is designed to preserve continence and achieve satisfactory quality of life. This study evaluated a comprehensive group of clinical and social outcomes.

**Methods:**

An international multicentre study from eight Nordic hospitals involving examination of case records and a patient‐reported questionnaire survey of all patients born with TCA between 1987 and 2006 was undertaken.

**Results:**

Of a total of 116 patients, five (4·3 per cent) had died and 102 were traced. Over a median follow‐up of 12 (range 0·3–33) years, bowel continuity was established in 75 (73·5 per cent) at a median age of 11 (0·5–156) months. Mucosectomy with a short muscular cuff and straight ileoanal anastomosis (SIAA) (29 patients) or with a J pouch (JIAA) (26) were the most common reconstructions (55 of 72, 76 per cent). Major early postoperative complications requiring surgical intervention were observed in four (6 per cent) of the 72 patients. In 57 children aged over 4 years, long‐term functional bowel symptoms after reconstruction included difficulties in holding back defaecation in 22 (39 per cent), more than one faecal accident per week in nine (16 per cent), increased frequency of defaecation in 51 (89 per cent), and social restrictions due to bowel symptoms in 35 (61 per cent). Enterocolitis occurred in 35 (47 per cent) of 72 patients. Supplementary enteral and/or parenteral nutrition was required by 51 (55 per cent) of 93 patients at any time during follow‐up. Of 56 responders aged 2–20 years, true low BMI for age was found in 20 (36 per cent) and 13 (23 per cent) were short for age.

**Conclusion:**

Reconstruction for TCA was associated with persistent bowel symptoms, and enterocolitis remained common. Multidisciplinary follow‐up, including continuity of care in adulthood, might improve care standards in patients with TCA.

## Graphical abstract

In a multicentre study, outcome of surgery for total colonic aganglionosis was evaluated in 116 patients. In long‐term follow‐up, patients with a J pouch reconstruction reported better faecal continence and those with a Duhamel reconstruction reported fewer episodes of enterocolitis. Nutritional support was needed extensively in all groups with various lengths of resected aganglionotic bowel.

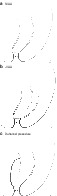

Problems persist into adulthood

## Abstract (es)

**Antecedentes:**

La cirugía de la aganglionosis colónica total (*total colonic aganglionosis*, TCA) está diseñada para preservar la continencia y lograr una calidad de vida satisfactoria. Este estudio evaluó un gran número de resultados clínicos y sociales.

**Métodos:**

Se realizó un estudio internacional multicéntrico en ocho hospitales nórdicos en el que se incluyeron las historias clínicas de todos los pacientes nacidos con TCA entre 1987 y 2006. Se invitó a los pacientes y a sus cuidadores a responder una encuesta sobre la función intestinal, el crecimiento y las necesidades nutricionales, así como la repercusión social de la enfermedad a largo plazo.

**Resultados:**

De un total de 116 pacientes, 5 (4,3%) habían fallecido y 102 respondieron la encuesta. Con una mediana de seguimiento de 12 años (rango 0,5–33), se había restablecido la continuidad intestinal en 75/102 (74%) a una mediana de edad de 11 meses (0,5‐156). La mucosectomía con un manguito muscular corto y anastomosis ileoanal directa (*short muscular cuff and straight ileo‐anal anastomosis*, SIAA) *n* = 29 o con reservorio en J (JIAA) *n* = 26 (55/72, 76%) fueron las reconstrucciones más habituales. Las complicaciones postoperatorias precoces que precisaron una intervención quirúrgica fueron muy poco frecuentes, pero se presentaron en 4/72 (5%) pacientes. Los síntomas a largo plazo relacionados con la función intestinal tras la reconstrucción, valorados en 57 niños mayores de cuatro años, fueron la dificultad para retener la defecación en 14 (25%), la encopresis en 21 (37%), las pérdidas fecales > 1/semana en 9 (16%) y el aumento de la frecuencia de defecación en 51 (89%). A largo plazo, se desarrolló una enterocolitis en 35/72 (47%) pacientes. Se precisó de nutrición enteral y/o parenteral suplementaria en algún momento del período de seguimiento en 51/93 (55%) pacientes. En los pacientes que respondieron a la encuesta entre 2 y 20 años (*n* = 56) de edad, se detectó un índice de masa corporal menor en 20 (35%) y una altura baja en 13 (23%) para su edad. En 35/57 (61%) pacientes mayores de 4 años con restauración del tránsito intestinal había restricciones sociales debido a los síntomas intestinales, de los que en 10 (17%) casos fueron moderadas o graves.

**Conclusión:**

La reconstrucción de una TCA se asocia con síntomas intestinales persistentes y la enterocolitis sigue siendo frecuente. Un seguimiento multidisciplinario, incluso en la edad adulta, podría mejorar los resultados en la cirugía de la TCA.

## Introduction

Total colonic aganglionosis (TCA) has an incidence of 1 in 50–100 000, affecting 2–5 per cent of all patients with Hirschsprung's disease^1^, ^2^. A few patients have aganglionosis that extends to the proximal small bowel or the entire small bowel^2^, ^3^, ^4^. TCA is a potentially life‐threatening condition with an overall mortality rate of 2–10 per cent due to obstructive ileus, short bowel syndrome, Hirschsprung‐associated enterocolitis (HAEC) and concomitant genetic syndromes^3^, ^4^, ^5^, ^6^, ^7^. After diagnosis, the length of normoganglionic bowel needs to be identified, and the bowel decompressed adequately. Definitive reconstruction is usually performed later, provided there is sufficient ganglionic small bowel available for restoration of bowel continuity with a reasonable likelihood of acceptable bowel function.

The most commonly performed surgical reconstructions in TCA are J pouch with ileoanal anastomosis (JIAA), straight ileoanal anastomosis (SIAA) and the Duhamel operation^3^, ^4^, ^5^, ^8^, ^9^, ^10^. The optimal age for reconstruction is not clear^4^, ^5^, ^11^, ^12^. All definitive surgical procedures in TCA aim to achieve faecal continence by preserving the sphincter musculature, even though the internal anal sphincter remains aganglionic and is a potential cause of obstructive defaecation and HAEC^13^, ^14^. In the long‐term, nutritional status and growth may be concerns, owing to loss of intestinal length, dysmotility and malabsorption^1^, ^15^, ^16^, ^17^. To date, no reconstructive surgical technique has emerged as superior to others with regard to functional outcomes or complications^3^, ^4^, ^18^, ^19^. The relative rarity of the condition and the lack of long‐term follow‐up have limited the conclusions that can be drawn from single‐institution series.

This study aimed to describe the experience of TCA in Nordic centres, including the evaluation of patient‐reported functional outcomes, HAEC, growth, nutrition, and the social impact of TCA extending into adulthood.

## Methods

This was a multicentre study within the Nordic Paediatric Surgery Study Consortium of eight paediatric surgical centres treating patients for TCA in Denmark, Finland, Norway and Sweden. The study covered a geographical area of 27 million inhabitants.

The study protocol was approved by the ethical committees or patient safety authorities of each participating centre, and nationwide for the Swedish centres. Participation was voluntary. Informed written consent was obtained from patients and/or their carers according to national legislation.

### Patient data

All patients born between 1987 and 2017 with TCA with or without small intestinal involvement were identified from hospital records. Baseline data, including sex, birthweight, gestational age, associated malformations or syndromes, surgical treatment and presence of HAEC, were obtained retrospectively. Survival and postoperative complications were graded according to the Clavien–Dindo classification^20^, ^21^.

### Patient‐reported outcomes

Patient‐reported outcomes were evaluated in a cross‐sectional manner using questionnaires mailed to the patients and/or caregivers. Patients were asked about their current weight and height, medication for bowel dysfunction, nutritional support, episodes of HAEC, fluid losses, obstructive symptoms, gas problems, and social impact on life. Patients aged 4 years or more with bowel continuity completed the Rintala bowel function score (BFS)^22^, ^23^, ^24^ with established normative values. Carers of patients under the age of 16 years assisted in answering the questionnaires.

### Operative management

**Fig. 1:**
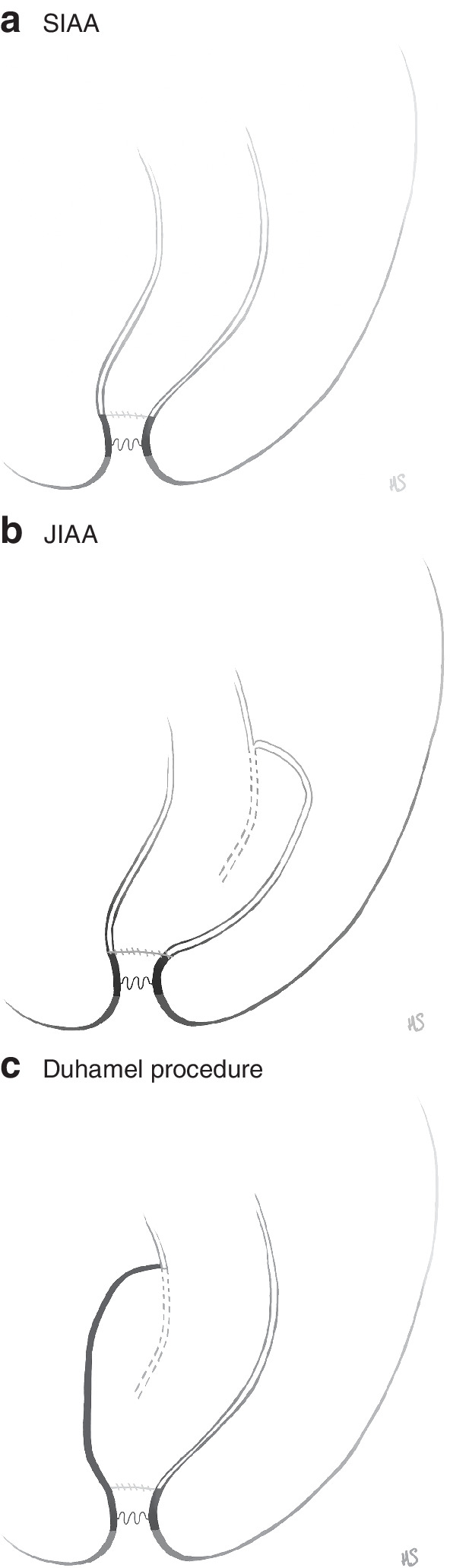
Reconstructive procedures for bowel continuity in total colonic aganglionosis **a** Straight ileoanal anastomosis (SIAA); **b** J pouch with ileoanal anastomosis (JIAA); **c** Duhamel procedure. Surgical reconstructive procedures for total colonic aganglionosis now being performed in the Nordic countries. Preparing for best possible functional outcome, the main overall intention in all three methods is to remove the aganglionotic bowel extensively, preserving the anal sphincter.

The level of aganglionosis was determined by assessment of full‐thickness biopsies at all centres. Decisions concerning the reconstructive method, timing of surgery and postoperative management were made independently by each centre or surgeon. The three main reconstructive procedures used during the whole study interval are illustrated in *Fig*. *1a–c*. Rehbein's procedure, performed only during a limited period, included open resection of the aganglionotic bowel and end‐to‐end anastomosis with a 3–5‐cm rectum stump.

Intrasphincteric botulinum toxin A injection was introduced in the Nordic countries in 2004, and used for the treatment of recurrent or persistent HAEC or obstructive symptoms^25^.

### Definitions

HAEC was defined on a clinical basis as a combination of diarrhoea and/or foul‐smelling stools, fever, and need for antibiotic treatment. Obstructive symptoms were defined as difficulty in passing stool despite soft faecal consistency and/or difficulty in passing gas. Terms regarding bowel function within the BFS were used according to the original BFS questionnaire. Difficulties in holding back was equivalent to faecal urgency, and the term ‘faecal accident’ was used to distinguish a more severe faecal leakage from soiling. Using the BFS (score 1–20, where 20 is best) for assessing the overall bowel function, normal function was defined as a score of 17 or above, moderate function as 12–16, and poor function as less than 12^22^, ^23^, ^24^. BMI for children and adults was calculated according to the WHO definition^26^, ^27^. More than two standard deviations below normal height and/or BMI was considered to be impaired growth.

### Statistical analysis

Continuous data are presented as median (range) and categorical data as frequencies. Fisher's exact test and the Fisher–Freeman–Halton exact probability test were used for comparison of categorical variables, and continuous variables were assessed with the Mann–Whitney *U* and Kruskal–Wallis rank sum tests. *Post hoc* testing was performed for multiple analyses. Spearman's rank correlation was used for correlation analyses of non‐parametric data. Statistical analysis was carried out with SPSS® version 24.0 software (IBM, Armonk, New York, USA). *P* < 0·050 was considered to be statistically significant.

## Results

In total, 116 patients with TCA (78 male, 67·2 per cent) were identified, corresponding to an incidence of one in 100 000 live births. The median follow‐up period was 12 (range 0·5–33) years. Patient characteristics are outlined in *Table* *1*. Data for time of diagnosis were available for 106 patients, of whom 70 (66·0 per cent) had been diagnosed within the first month, 81 (76·4 per cent) within 3 months, and 101 (95·3 per cent) within 1 year of birth. During the follow‐up period, five patients had died, from complications of short bowel syndrome (2), intestinal or liver–intestinal transplantation (1) or associated syndromes (2). Co‐morbidities are shown in *Table* *2*.

**Table 1:** Patient characteristics and surgical interventions

	*n*	No. of patients
**Gestational age (weeks)** *	98	40 (29–43)
**Sex ratio (F** : **M)**	116	38 : 78
**Birthweight (g)** *	101	3410 (1410–5000)
**Family history of disease**	113	21 (18·6)
**Syndrome**	108	21 (19·4)
**Associated congenital anomalies**	108	17 (15·7)
**Age at diagnosis (biopsy) (days)** *	102	20 (1–1680)
**Presenting sign**		
Obstruction	105	86 (81·9)
Enterocolitis	105	6 (5·7)
Perforation	105	9 (8·6)
Constipation	105	5 (4·8)
> 1 sign	105	9 (8·6)
Missing	116	11 (9·5)
**Age at restoration of bowel continuity (months)** *	70	11 (0·3–156)
**Primary ostomy**	116	102 (87·9)
**Colectomy before reconstruction**	113	44 (38·9)
**Completed reconstruction for bowel continuity**	102	75 (73·5)
Straight ileoanal anastomosis	75	29 (39)
J pouch–ileoanal anastomosis	75	26 (35)
Duhamel procedure	75	11 (15)
Rehbein procedure	75	6 (8)
Unknown procedure	75	3 (4)
**Ostomy at time of survey**	102	27 (26·5)
**Intestinal transplantation**	102	4 (3·9)
**No data available for timing or type of reconstruction**	102	2 (2·0)
**Overall survival**	116	111 (95·7)

Values in parentheses are percentages unless indicated otherwise;

* values are median (range).

**Table 2:** Co‐morbidity in patients with total colonic aganglionosis

	No. of patients (*n* = 108)
**Any co‐morbidity**	29 (26·9)
**Syndrome**	21 (19·4)
Trisomy 21 (Down syndrome)	10 (9·3)
Shah–Waardenburg syndrome	3 (2·8)
Haddad syndrome	2 (1·9)
Cartilage–hair hypoplasia	2 (1·9)
Marcus Gunn phenomenon	2 (1·9)
McKusick–Kaufman syndrome	1 (0·9)
Kabuki syndrome	1 (0·9)
**Concomitant malformation**	19 (17·6)
Cardiac	6 (5·6)
Gastrointestinal	4 (3·7)
Small bowel atresia	2 (1·9)
Duodenal atresia	2 (1·9)
Genitourinary	8 (7·4)
Single kidney	4 (3·7)
Dysplastic kidney	1 (0·9)
Congenital hydronephrosis	1 (0·9)
Micropenis	1 (0·9)
Hypospadias	1 (0·9)
Cleft palate	1 (0·9)
Other	1 (0·9)
**Concomitant disease**	9 (8·3)
Hypothyroidism	2 (1·9)
Coeliac disease	2 (1·9)
Ganglioneuroblastoma	1 (0·9)
Ganglioneuroma	1 (0·9)

Values in parentheses are percentages.

### Operative details

The length of resected small bowel was documented in 94 patients: 0–10 cm of ileum in 39 (41 per cent), 11–20 cm of ileum in 30 (32 percent), 21–50 cm of ileum in ten (11 per cent), 51–100 cm of ileum–jejunum in eight (9 per cent), and more than 100 cm of small bowel in seven (7 per cent). Four patients (4 per cent) had total/near total intestinal aganglionosis. Data regarding bowel reconstructions are shown in *Table* *1*. Four (3·9 per cent) of 102 patients had undergone intestinal transplantation owing to total/near total intestinal aganglionosis, three of whom were alive with functioning grafts after 8, 3 and 2 years. Of 102 patients for whom data were available, 75 (73·5 per cent) had undergone surgery to restore intestinal continuity and 72 had type of procedure documented. The most common reconstructive technique was mucosectomy with a short muscular cuff and an ileoanal anastomosis (IAA), which was performed in 55 (76 per cent) of the 72 patients, either as a straight IAA (SIAA) (29 patients) or with a J pouch (JIAA) (26). Definitive reconstructions were performed at 0–6 months of age in 26 (37 per cent), at 6–12 months in 14 (20 per cent), at 1–3 years in 21 (30 per cent), and at 3 years or older in nine (13 per cent) (data available for 70 of the 75 patients). All children who had reconstructions between 0 and 6 months of age had aganglionosis confined to 20 cm or less of small bowel.

Reoperation for residual aganglionosis was required in five (4·9 per cent) of the 102 patients overall: three redo reconstructions, one conversion to a permanent stoma and reresection, and a more proximal stoma in one patient with a permanent stoma. Surgical intervention for adhesive obstruction or volvulus was performed in eight patients (7·8 per cent). Some 27 of these 102 patients had an enterostomy, which required 33 operations in 12 of these patients, mostly (28 of 33) for prolapse.

### Postoperative complications after restoration of bowel continuity

Postoperative complications within 4 weeks of restoration of bowel continuity for the main reconstructive procedures are shown in *Table* *3*. Major early complications (Clavien–Dindo grade IIIb or above) requiring surgical intervention occurred in four (6 per cent) of the 72 patients (3 anastomotic leaks, 1 severe anastomotic stricture), all in patients without a covering enterostomy at the time of reconstruction. No life‐threatening complications or deaths occurred within the first 30 days after reconstruction.

**Table 3:** Postoperative complications during the first 4 weeks after restoration of bowel continuity, and occurrence of Hirschsprung‐associated enterocolitis during follow‐up

	All (*n* = 72)	SIAA (*n* = 29)	JIAA (*n* = 26)	Duhamel (*n* = 11)	Rehbein (*n* = 6)
**Covering enterostomy**	37 (51)	5 (17)†	23 (88)	9 (82)	0 (0)
**Clavien–Dindo grade of short‐term complication**	25 (35)	10 (34)	8 (31)	5 (45)	2 (33)
I	14 (19)	7 (24)	6 (23)	1 (9)	0 (0)
II	2 (3)	1 (3)	0	1 (9)	0 (0)
IIIa	5 (7)	1 (3)	0	2 (18)	2 (33)
IIIb	4 (6)	1 (3)	2 (8)	1 (9)	0 (0)
IV	0 (0)	0 (0)	0 (0)	0 (0)	0 (0)
V	0 (0)	0 (0)	0 (0)	0 (0)	0 (0)
**HAEC**	35 (49)	15 (52)	13 (50)	4 (36)	3 (50)
1–3 episodes	15 (21)	5 (17)	5 (19)	2 (18)	3 (50)
Recurrent HAEC*	20 (28)	10 (34)	8 (31)	2 (18)	0 (0)

Values in parentheses are percentages.

* More than three episodes or chronic symptoms of Hirschsprung‐associated enterocolitis (HAEC). SIAA, straight ileoanal anastomosis; JIAA, J pouch–ileoanal anastomosis.

† *P* ≤ 0·003 *versus* JIAA and Duhamel procedure (Fisher's exact test); all other comparisons were not significant.

A covering enterostomy significantly reduced the incidence of early postoperative complications (7 of 37 *versus* 17 of 35 without a covering enterostomy; *P* = 0·006). Protective stomas were closed a median of 2·5 (range 1–12) months after the reconstruction. The most frequent early complication among patients without a protective stoma was intense perianal rash in 40 per cent (14 of 35). There was no difference in the incidence of early complications between the main three procedures: SIAA, JIAA and the Duhamel operation (*P* = 1·000).

There were no significant differences in the prevalence of long‐term complications between procedures (*P* = 1·000). Eight of 72 patients (11 per cent) had a stoma re‐established at a median of 2 (range 0·1–5) years after reconstruction. Reasons for stoma formation were a combination of severe obstruction and diarrhoea, with severe perianal excoriation in seven patients and anal pain in one.

### Hirschsprung‐associated enterocolitis after bowel reconstruction

Based on chart review, the overall rate of HAEC among reconstructed patients was 47 per cent (35 of 75) (*Table* *3*). Twenty‐five per cent of patients (18 of 72) were treated at least once with intrasphincteric botulinum toxin A injection, owing to outlet obstruction in six patients, HAEC in eight, and a combination of these in four patients. All but one of these 18 patients had undergone SIAA or JIAA.

### Growth and nutritional status

Growth and nutritional needs were assessed from the charts of 94 patients. Overall, 44 patients (47 per cent) had required supplementary nutritional support owing to insufficient growth or malnourishment. Parenteral nutrition had been administered in the past in 31 patients (33 per cent) up to a median age of 3 (range 0·8–6) years. At the time of follow‐up, 14 patients (15 per cent) used parenteral support on a regular basis, nine had a gastrostomy for nutritional supplementation at follow‐up, and six had previously had a gastrostomy.

### Patient‐reported functional outcomes

#### Bowel function

**Fig. 2:**
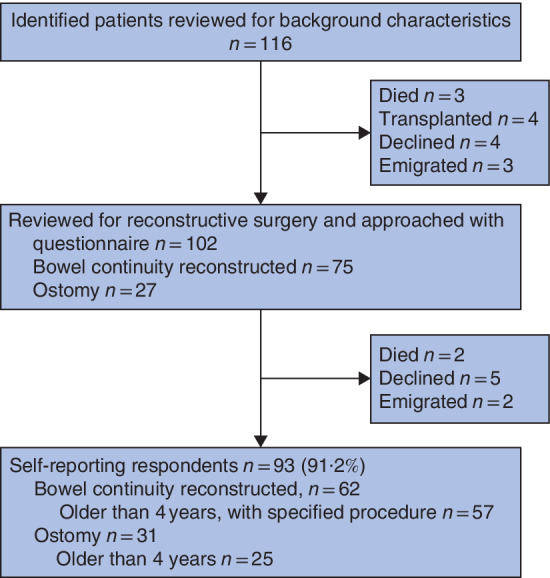
Flow diagram for the study

Some 102 patients were traced for the functional outcomes survey and 93 completed questionnaires were returned (91·2 per cent response rate) (*Fig*. *2*). Bowel function was assessed in 57 patients older than 4 years and with native bowel continuity and known reconstructive technique at a median age of 10 (range 0·5–156) months. In all patients, less than 60 cm of small bowel had been resected. The age distribution and length of resected small bowel did not differ between patients operated on by SIAA, JIAA or the Duhamel procedure.

Patient‐reported outcomes for continence after reconstruction are shown in *Table* *4*. No significant differences in continence parameters were observed between procedures, apart from better perceived ability to hold back defaecation after JIAA than SIAA or Duhamel (*P* = 0·007). Increased frequency of defaecation (more than twice daily) affected 86–100 per cent of patients in the long term (median 4 (range 3–10) bowel motions per day). Social problems due to bowel function were common and reported by 35 (61 per cent) of 57 patients, perceived as moderate or severe by ten patients (18 per cent). Long‐term outcomes according to the BFS were good or normal (BFS 17–20) in 13 patients (23 per cent), moderate (BFS 16–12) in 33 (58 per cent) and poor (BFS less than 12) in 11 (19 per cent), with no differences between procedures.

**Table 4:** Patient‐reported bowel symptoms in 57 children with total colonic aganglionosis aged 4 years or more, stratified by surgical technique for restoring bowel continuity

	All (*n* = 57)	SIAA (*n* = 21)	JIAA (*n* = 22)	Duhamel (*n* = 9)	Rehbein (*n* = 5)
**Ability to hold back defaecation**					
Always	35 (61)	10 (48)	19 (86)†	3 (33)	3 (60)
Problems < 1/week	15 (26)	7 (33)	1 (5)	5 (56)	2 (40)
Weekly problems	5 (9)	4 (19)	1 (5)	0	0 (0)
No control	2 (4)	0	1 (5)	1 (11)	0 (0)
**Feels urge to defaecate (rectal sensation)**					
Always	19 (33)	6 (29)	7 (32)	5 (56)	1 (20)
Most of the time	16 (28)	8 (38)	5 (23)	1 (11)	2 (40)
Uncertain	8 (14)	3 (14)	3 (14)	1 (11)	1 (20)
Never	14 (25)	4 (19)	7 (32)	2 (22)	1 (20)
**Frequency of bowel movements**					
Every other day to twice a day	6 (11)	3 (14)	2 (9)	1 (11)	0 (0)
Less often	0 (0)	0 (0)	0 (0)	0 (0)	0 (0)
More often	51 (89)	18 (86)	20 (91)	8 (89)	5 (100)
**Soiling**					
Never	12 (21)	5 (24)	4 (18)	1 (11)	2 (40)
< 1/week	24 (42)	6 (29)	10 (45)	5 (56)	3 (60)
≥ 1/week	18 (32)	9 (43)	6 (27)	3 (33)	0 (0)
Always/daily	3 (5)	1 (5)	2 (9)	0 (0)	0 (0)
**Faecal accidents**					
Never	32 (56)	9 (43)	13 (59)	7 (78)	3 (60)
< 1/week	16 (28)	6 (29)	7 (32)	2 (22)	1 (20)
≥ 1/week	8 (14)	5 (24)	2 (9)	0 (0)	1 (20)
Always/daily	1 (2)	1 (5)	0 (0)	0 (0)	0 (0)
**Constipation**					
No treatment	54 (95)	19 (90)	22 (100)	8 (89)	5 (100)
Treated by diet	0 (0)	0 (0)	0 (0)	0 (0)	0 (0)
Medically treated	1 (2)	1 (5)	0 (0)	0 (0)	0 (0)
Enemas	2 (4)	1 (5)	0 (0)	1 (11)	0 (0)
**Social problems**					
None	22 (39)	12 (57)	8 (36)	1 (11)	1 (20)
Mild	25 (44)	6 (29)	12 (55)	6 (67)	1 (20)
Moderate	7 (12)	1 (5)	2 (9)	1 (11)	3 (60)
Severe	3 (5)	2 (10)	0 (0)	1 (11)	0 (0)
**BFS score** *	15 (5–19)	14·5 (5–19)	16 (8–19)	14 (11–17)	15 (12–18)

Values in parentheses are percentages unless indicated otherwise;

* values are median (range). SIAA, straight ileoanal anastomosis; JIAA, J pouch–ileoanal anastomosis; BFS, bowel function score (1–20).

† *P* = 0·007 *versus* SIAA and Duhamel procedure (Kruskal–Wallis test); all other comparisons were not significant.

In a specific analysis of outcomes among adolescents and adults, in 21 patients of median age 26 (range 15–33) years, continence abnormalities lasting for more than 1 week involved: difficulty holding back defaecation in two patients (10 per cent); soiling in three (14 per cent); faecal accidents in two (10 per cent); poor or absent rectal sensation in 11 (52 per cent); increased bowel frequency in 20 (95 per cent); and constipation in one patient (5 per cent). Overall, continence abnormalities were not significantly different from those in the younger cohort (*P* = 0·311). Six patients had social problems that were severe or restricted social activities.

#### Episodes of Hirschsprung‐associated enterocolitis and other abdominal symptoms

At follow‐up, 22 (39 per cent) of 57 patients reported having ever experienced at least one episode of HAEC; 12 (21 per cent) had experienced HAEC during the past year: eight after JIAA, four after SIAA, and none after the Duhamel procedure (*P* = 0·007). Recurrent or chronic HAEC requiring medical treatment was reported by 22 (39 per cent) of the 57 patients overall: 12 of 21 patients after SIAA, ten of 22 after JIAA, and none after the Duhamel operation (*P* = 0·003). Botulinum toxin A injections for recurrent HAEC or obstructive symptoms had been required by 25 per cent overall (14 of 57): three of 21 after SIAA, ten of 22 after JIAA, and one of nine after the Duhamel procedure (*P* = 0·110). Of 27 respondents with a permanent enterostomy, 14 had required treatment for HAEC, with seven reporting recurrent HAEC. This level of HAEC did not differ significantly from that seen in patients with bowel continuity (*P* = 0·234). Other abdominal symptoms – bloating/distension and involuntary loss of gas more than once a week – were each reported by 15 of 57 patients with bowel continuity, and by three and seven patients respectively of the 27 patients with an enterostomy.

#### Satisfaction with bowel function

On a Likert scale of 1–5 (5, very satisfied; 1, very unsatisfied), 28 (49 per cent) of 57 patients with bowel continuity were very or mostly satisfied with their bowel function (score 4–5), 15 (26 per cent) were moderately satisfied (score 3), and 14 (25 per cent) were mostly or very unsatisfied, with no significant difference between procedures. Among 25 patients with a stoma present for more than 4 years, five were very or mostly satisfied, 16 were moderately satisfied, and four were mostly or very unsatisfied with their bowel functional status (*P* = 0·130 *versus* patients with a bowel reconstruction).

### Medication

Of 93 respondents, 53 (57 per cent) reported using medication regularly for bowel symptoms. Antibiotics for HAEC were the most frequent medical treatment reported: 24 (26 per cent) used regular treatment with metronidazole in combination with trimethoprim–sulfamethoxazole, oral vancomycin or amoxicillin, and in addition two patients (2 per cent) included fluconazole in their HAEC regimen. Loperamide was used by 21 patients (23 per cent), probiotics by seven (8 per cent), and proton pump inhibitors by five (5 per cent).

### Growth and nutritional needs

**Fig. 3:**
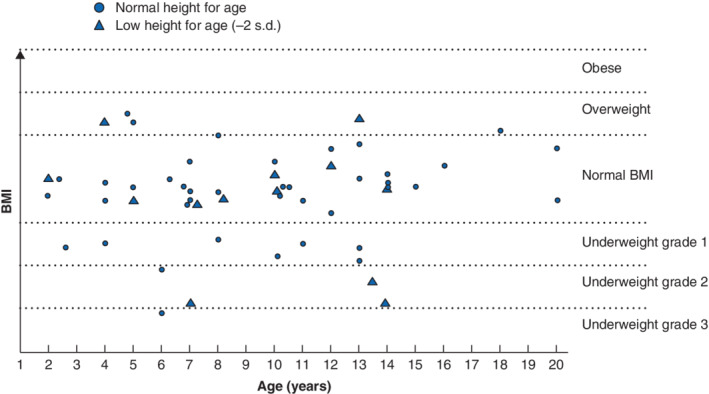
BMI in 56 patients aged 2–20 years with total colonic aganglionosis

Among 93 survey respondents, 37 (40 per cent) reported having, or having had, a growth problem (*Table* *5*). Of 56 responding patients aged 2–20 years, 13 (23 per cent) had low height for age, and 12 (21 per cent) had low BMI for age (more than −2 s.d. below normal height or BMI). Three (5 per cent) of these 56 patients had both low BMI and low height for age. Although eight patients (14 per cent) had an apparently normal BMI for height, their height for age was low. Consequently, 20 (36 per cent) of the 56 patients overall were defined as having a true low BMI (*Fig*. *3*). Half of the 20 patients with low BMI had not received any nutritional support. Among 20 respondents aged over 20 years, BMI was normal in 15 and five were overweight. No patient in this group was underweight, but height was below −2 s.d. in three men. None of the 20 patients in the older age group had a bowel resection extending longer than 55 cm, compared with eight (11 per cent) of 73 patients in the younger age group (*P* = 0·194).

**Table 5:** Patient‐reported growth and nutritional needs for 93 patients with total colonic aganglionosis of median age 9 (range 0·5–33) years, categorized by length of resected distal small bowel

		Length of small bowel resected (cm) (*n* = 83)				
	Overall (*n* = 93)	0–10 (*n* = 34)	11–20 (*n* = 26)	21–60 (*n* = 13)	> 60 (*n* = 10)	*P* #
**Growth problems** *						
Yes	37 (40)	8 (24)	11 (42)	7 (54)	6 (60)	0·046
**Enteral nutritional support**						0·001
Never	37 (40)	20 (59)	12 (46)	2 (15)	1 (10)	
Any†	56 (60)	14 (41)	14 (54)	11 (85)	9 (90)	
**Parenteral nutrition** **‡**						< 0·001
Never	34 (37)	17 (50)	12 (46)	2 (15)	0 (0)	
Any†	58 (63)	17 (50)	14 (54)	11 (85)	10 (100)	
**Iron supplement** **‡**						< 0·001
Never	57 (62)	27 (79)	16 (62)	2 (15)	3 (30)	
Any†	35 (38)	7 (21)	10 (38)	11 (85)	7 (70)	
**Vitamin B**_**12**_ **supplement** **‡**						< 0·001
Never	64 (70)	29 (85)	20 (77)	1 (8)	2 (20)	
Yes†	28 (30)	5 (15)	6 (23)	12 (92)	8 (80)	
Regularly	20 (22)					
**Sodium chloride supplement**						< 0·001
Never	43 (46)	22 (65)	14 (54)	2 (15)	0 (0)	
Any†	50 (54)	12 (35)	12 (46)	11 (85)	10 (100)	
**Rehydration solution**						0·025
Never	53 (57)	21 (62)	18 (69)	5 (38)	3 (30)	
Any†	40 (43)	13 (38)	8 (31)	8 (62)	7 (70)	

Values in parentheses are percentages.

* More than −2 s.d. below normal height and/or BMI.

† Includes the responses ‘previously’, ‘sometimes’ and ‘regularly’.

‡ One patient did not respond.

# *P* value for 0–20 cm *versus* ≥ 21 cm resected (Fisher's exact test).

The use of supplementary nutrition among respondents is shown in *Table* *5*. Overall, supplementary enteral and/or parenteral nutrition had been used by 51 (55 per cent) of 93 respondents, and 33 (35 per cent) currently received parenteral nutrition either regularly or occasionally. Although the requirement for this supplementary nutrition and supplementary iron, vitamin B_12_, sodium chloride and rehydration solutions was greater among patients with more than 20 cm of small bowel resected (77–92 per cent), requirements among patients with 0–20‐cm resections were also common (19–48 per cent) (*Table* *5*).

## Discussion

This study, spanning the past 30 years and a geographical area of 27 million inhabitants, examined long‐term operative and patient‐reported outcomes for TCA. The overall mortality rate of 4·3 per cent (5 of 116) was lower than that reported in previous series (6–19 per cent)^4^, ^6^, ^19^. This may reflect access to treatment and follow‐up in the Nordic countries. Deaths were caused by associated co‐morbidities or complete aganglionosis of the bowel and, in contrast to other series^4^, ^7^, ^13^, ^14^, were not directly attributable to HAEC. The overall co‐morbidity rate of 26·9 per cent was higher than that in previous reports (4–16 per cent)^1^, ^7^, ^16^, and could be attributed to routine screening practices for associated malformations at tertiary neonatal surgical centres. Nearly all patients with TCA (95·3 per cent) were diagnosed within the first year of life, and most (76·4 per cent) within the first 3 months.

In the Nordic countries, IAA with or without a J pouch comprised the large majority of the reconstructions (76 per cent). As with previous reports of operative techniques for Hirschsprung's disease^3^, ^4^, ^8^, ^18^, ^19^, no significant differences were found in early or long‐term complications between the different surgical techniques. The low incidence of major early complications requiring surgical intervention (Clavien–Dindo grade IIIb), the low need for repeat reconstruction owing to residual aganglionosis (4·9 per cent), and the absence of life‐threatening complications supports the safety and appropriateness of managing TCA at tertiary centres. A protective stoma at the time of reconstruction conferred a protective effect from early complications; all three anastomotic leaks after reconstruction were among patients without a covering stoma.

Based on chart review, the overall risk of HAEC after restoration of bowel continuity was 47 per cent among patients with TCA, reported elsewhere^1^, ^4^, ^7^, ^13^, ^14^ to be in the range of 42–55 per cent. In the present study, recurrent or chronic HAEC and the need for intrasphincteric botulinum injections were less common after the Duhamel operation than after SIAA or JIAA, but patient numbers after the Duhamel operation (11) were small.

For long‐term bowel functional outcomes, 23 per cent of patients achieved a good or normal BFS, and 51 per cent reported subjective satisfaction on the Likert scale. A high proportion of patients (57 per cent) used adjunctive medication, including antibiotics and loperamide, to control bowel symptoms. Nonetheless, significant continence issues, including difficulties in holding back faeces, soiling and accidents, were common, consistent with other studies of TCA^1^, ^6^, ^7^. Although better ability to hold back defaecation was reported by patients managed by JIAA reconstruction than for the other procedures, this was not reflected as any difference in the overall prevalence of soiling or accidents by procedure. Diminished ability to hold back is likely to reflect decreased anal sensation, rapid bowel movements and absence of the rectoanal inhibitory reflex^11^, ^28^, ^29^. In the present cohort, the overall proportion with frequent bowel movements was 89 per cent, with 37 per cent reporting uncertain or absent rectal sensation. Unlike series of endorectal pull‐through procedures for predominantly short‐segment Hirschsprung's disease^30^ or a recent national Swedish series of TCA^1^, significant reductions in the prevalence of abnormalities in neorectal sensation or other bowel symptoms among older patients (aged 15–33 years) compared with younger patients were not seen. None of the adult patients had a resection greater than 55 cm, whereas this was the case for several children. In the absence of a change in phenotype of TCA over the years, the skewed distribution might indicate that those patients had not been operated on in the past or were not identified for inclusion in the study if they had been treated at other departments.

Adequately addressing social morbidity related to bowel function is an important concern for follow‐up among patients with Hirschsprung's disease^17^, ^31^. Of patients with TCA and bowel continuity, major restrictions of social life or severe social/psychological problems due to bowel problems were identified in 18 per cent, but occasional social limitations were reported by a further 44 per cent. In addition to continence issues or frequent bowel movements, these may relate to episodes of involuntary gas loss and abdominal bloating, which were relatively common. Psychological morbidity may also result from the repeated hospital admissions and surgical procedures that patients with TCA endure during their lifetime.

Reports on the nutritional needs of patients with TCA are sparse^1^, ^15^, ^16^. The present study identified that supplementary nutrition was required commonly, even among patients with 0–20 cm of small bowel resected. Overall, 40 per cent of patients reported having experienced growth problems, and 36 per cent of children (20 of 56) and 15 per cent of adults (3 of 20) had a low BMI or were below 2 s.d. in height. Half of the patients who had not received nutritional supplements reported growth issues, indicating that enhanced attention to patients' nutritional needs by a multidisciplinary team is likely to be beneficial.

Owing to the relative rarity of TCA, a limitation of this study is the size of available cohorts, particularly with regard to comparison of outcomes between different surgical procedures. The cross‐sectional study design meant that longitudinal changes could not be addressed.

Persistent bowel symptoms and enterocolitis remain common long‐term concerns after successful reconstruction for TCA. As the condition poses lifelong problems, postoperative follow‐up should encompass wider attention to the nutritional and psychosocial status of the condition. Ensuring robust arrangements for transition from the paediatric setting into adult care is essential.
